# Clinical advances of RNA therapeutics for treatment of neurological and neuromuscular diseases

**DOI:** 10.1080/15476286.2022.2066334

**Published:** 2022-04-28

**Authors:** Anja Holm, Stine N. Hansen, Henrik Klitgaard, Sakari Kauppinen

**Affiliations:** a Center for RNA Medicine, Department of Clinical Medicine, Aalborg University, A.C. Meyers Vænge 15, 2450 Copenhagen, Denmark; b Neumirna Therapeutics, A.C. Meyers Vænge 15, 2450 Copenhagen, Denmark

**Keywords:** Small interfering RNA, antisense oligonucleotide, RNA-based therapeutics, CNS, neurological disease, gene silencing, clinical trial, neuromuscular disorder

## Abstract

RNA therapeutics comprise a diverse group of oligonucleotide-based drugs such as antisense oligonucleotides (ASOs), small interfering RNAs (siRNAs), and short hairpin RNAs (shRNAs) that can be designed to selectively interact with drug targets currently undruggable with small molecule-based drugs or monoclonal antibodies. Furthermore, RNA-based therapeutics have the potential to modulate entire disease pathways, and thereby represent a new modality with unprecedented potential for generating disease-modifying drugs for a wide variety of human diseases, including central nervous system (CNS) disorders. Here, we describe different strategies for delivering RNA drugs to the CNS and review recent advances in clinical development of ASO drugs and siRNA-based therapeutics for the treatment of neurological diseases and neuromuscular disorders.

**Abbreviations** 2’-MOE: 2’-*O*-(2-methoxyethyl); 2’-*O*-Me: 2’-*O*-methyl; 2’-F: 2’-fluoro; AD: Alzheimer's disease; ALS: Amyotrophic lateral sclerosis; ALSFRS-R: Revised Amyotrophic Lateral Sclerosis Functional Rating Scale; ARC: Antibody siRNA Conjugate; AS: Angelman Syndrome; ASGRP: Asialoglycoprotein receptor; ASO: Antisense oligonucleotide; AxD: Alexander Disease; BBB: Blood brain barrier; Bp: Basepair; CNM: Centronuclear myopathies; CNS: Central Nervous System; CPP: Cell-penetrating Peptide; CSF: Cerebrospinal fluid; DMD: Duchenne muscular dystrophy; DNA: Deoxyribonucleic acid; FAP: Familial amyloid polyneuropathy; FALS: Familial amyotrophic lateral sclerosis; FDA: The United States Food and Drug Administration; GalNAc: N-acetylgalactosamine; GoF: Gain of function; hATTR: Hereditary transthyretin amyloidosis; HD: Huntington's disease; HRQOL: health-related quality of life; ICV: Intracerebroventricular; IT: Intrathecal; LNA: Locked nucleic acid; LoF: Loss of function; mRNA: Messenger RNA; MS: Multiple Sclerosis; MSA: Multiple System Atrophy; NBE: New Biological Entity; NCE: New Chemical Entity; NHP: Nonhuman primate; nt: Nucleotide; PD: Parkinson's disease; PNP: Polyneuropathy; PNS: Peripheral nervous system; PS: Phosphorothioate; RISC: RNA-Induced Silencing Complex; RNA: Ribonucleic acid; RNAi: RNA interference; s.c.: Subcutaneous; siRNA: Small interfering RNA; SMA: Spinal muscular atrophy; SMN: Survival motor neuron; TTR: Transthyretin

## Introduction

Development of medicines for neurological diseases has traditionally been driven by phenotypic drug discovery approaches and improved understanding of key neurotransmitter and receptor systems including γ-aminobutyric acid (GABA)[1] and dopamine[2] as well as voltage-operated ion channels like Na^+^ and Ca^2+^ channels[3]. This has resulted in the discovery of numerous new chemical entities (NCEs) for neurological diseases, when combined with advances in drug discovery technologies such as high throughput screening, computer modelling, and combinatorial chemistry. Indeed, NCEs have provided new symptomatic treatments for a wide variety of neurological diseases with improved tolerability and reduced potential for drug-drug interactions. The last two decades have also witnessed a major surge in the discovery and development of potential monoclonal antibody-based therapies within neurological diseases, named as new biological entities (NBEs). A key outcome has been the identification of several development candidates pursuing the relevance of the aggregated protein theory in neurodegenerative diseases by reducing brain accumulation of β-amyloid or phosphorylated tau in Alzheimer's disease[4] or α-synuclein in Parkinson's disease[5].

Despite the progress in drug discovery efforts deploying NCE and NBE approaches, patients with neurological diseases still face major unmet needs due to the limited efficacy of symptomatic treatments, drug resistance, and absence of preventive and disease-modifying treatments. Gene therapy represents an interesting modality for a restricted number of patients within neurological diseases by providing a maintained supply of therapeutic proteins in patients with genetic disorders and certain rare neurological diseases[6]. However, gene therapies do not offer viable treatment options for neurological patients suffering from sporadic diseases. In contrast, RNA-based therapeutics can selectively interact with drug targets that are currently undruggable with NCE and NBE approaches and are capable of modulating entire disease pathways, thereby representing a new modality with unprecedented potential for generating disease-modifying drugs for neurological and neuromuscular diseases[7].

RNA therapeutics comprise a highly diverse group of oligonucleotide-based drugs, including antisense oligonucleotides (ASOs), small interfering RNAs (siRNAs), short hairpin RNAs (shRNAs), aptamers, microRNA inhibitors (antimiRs), and microRNA mimics[8]. Therapeutic ASOs and siRNAs represent the most clinically advanced RNA drug platforms, and as of today eight ASO and four siRNA drugs have been approved by the FDA and/or EMA for treatment of various diseases[9]. In this review, we focus on the use of ASOs and siRNAs to therapeutically modulate the expression of targets implicated in the pathogenesis of CNS diseases. Furthermore, we review potential approaches for delivering ASOs and siRNAs to the CNS and discuss recent advances in the clinical development of ASO- and siRNA-based therapeutics for the treatment of neurological diseases and neuromuscular disorders.

## Antisense oligonucleotides

**Figure 1.**
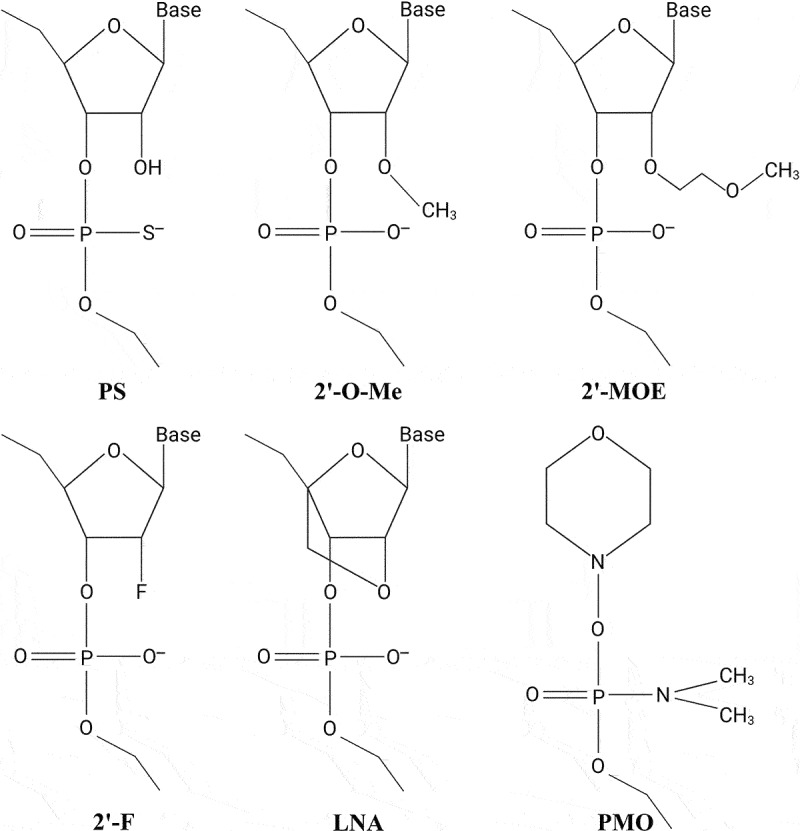
Common chemical modifications used in RNA-based therapeutics. Nuclease resistance of ASOs is improved by substituting the parent phosphodiester bonds with phosphorothioate (PS) linkages in which a sulphur atom replaces one of the non-bridging oxygen atoms in the phosphate group. In addition, PS modifications enhance plasma protein binding and reduce clearance of the ASOs, thereby enhancing the PK properties of PS-ASOs. Improved stability and increased binding affinity is achieved using nucleotides with sugar modifications, including different 2’-modified sugars such as the 2’-*O*-methyl (2’-*O*-Me), 2’-*O*-methoxyethyl (2’-MOE), or 2ʹ-fluoro (2’-F) modifications, or the bicyclic locked nucleic acid (LNA) modification, in which the ribose sugar is locked in a C3’-endo conformation by introduction of a 2’-O,4’-C methylene bridge. PMOs contain a backbone of hexagonal morpholine rings linked by phosphorodiamidate bonds.

ASOs are single-stranded oligonucleotides, typically 14–20 nucleotides (nt) in length, designed to bind and inhibit the functions of complementary RNA transcripts[10,11]. Most ASOs are chemically modified to increase binding affinity, improve nuclease resistance, and enhance pharmacokinetic (PK) properties of therapeutic ASOs *in vivo* (Figure. 1). Nuclease resistance of ASOs is markedly improved by substituting the parent phosphodiester bonds with phosphorothioate (PS) linkages in which a sulphur atom replaces one of the non-bridging oxygen atoms in the phosphate group. In addition, PS modifications enhance plasma protein binding and reduce clearance of the ASOs by glomerular filtration and urinary excretion, thereby enhancing the PK properties and facilitating delivery of PS-ASOs into many peripheral tissues[11,12]. PS modifications do not affect RNase H activity[11], but each PS substitution creates a stereocenter, thus giving rise to diastereomers potentially affecting potency and target affinity of the PS-modified ASO[12].

Improved stability and increased binding affinity is achieved using nucleotides with sugar modifications, including different 2’-modified sugars such as the 2’-O-methyl (2’-O-Me), 2’-O-methoxyethyl (2’-MOE), or 2ʹ-fluoro (2’-F) modifications, or conformationally restricted nucleotides such as the bicyclic locked nucleic acid (LNA) modification, in which the ribose sugar is locked in a C3’-endo conformation by introduction of a 2’-O,4’-C methylene bridge[13]. Nuclease resistance can also be enhanced by using peptide nucleic acids (PNAs) or phosphorodiamidate morpholino oligomers (PMOs), respectively. PNAs are uncharged oligonucleotide analogues, in which the sugar-phosphate backbone has been replaced by a peptide-like backbone consisting of N-(2-aminoethyl)-glycine units, whereas PMOs contain a backbone of hexagonal morpholine rings linked by phosphorodiamidate bonds[14,15]. However, both PNAs and PMOs exhibit rapid renal clearance and poor cellular uptake[16,17].

**Figure 2.**
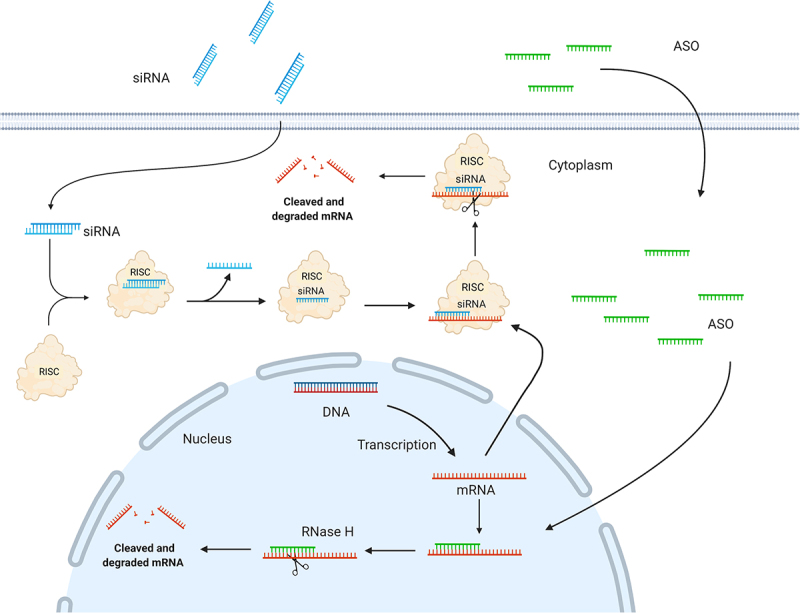
Schematic illustration depicting siRNA and ASO mechanisms of action. A: In the cytoplasm, siRNA triggers are incorporated into the RNA-induced silencing complex (RISC); a ribonucleoprotein complex consisting of Dicer, the RNA binding protein (TRBP), and the RNase Argonaute 2 (AGO2). Upon RISC loading, the strand with the less thermodynamically stable 5’-end is incorporated and guides the RISC to the complementary target mRNA. The mRNA target dissociates from the intact siRNA after AGO2 cleavage, freeing RISC to regenerate and cleave additional mRNA targets. B: ASOs modulate target mRNA expression either by steric blocking (mixmers) or recruitment of RNase H (gapmers). Gapmers are composed of a central DNA region flanked at both ends by chemically modified ribonucleotides and recruit RNase H, which recognizes and cleaves DNA:RNA heteroduplexes.

Two different design paradigms are used for ASOs: (i) gapmers for gene knockdown, in which a central deoxynucleotide region is flanked at both ends by modified ribonucleotides and, (ii) mixmers that serve as steric blockers for microRNA (miRNA) inhibition or splice modulation. Gene knockdown is mediated by RNase H, which binds and cleaves RNA/DNA heteroduplexes primarily in the nucleus (Fig. 2), whereas miRNA inhibition and splice modulation rely on steric blocking of the miRNA guide strand or specific splice sites, respectively, without causing RNA degradation[11].

### RNA interference

The endogenous RNA interference mechanism was discovered by Fire and Mello in 1998[18] and can be engaged by synthetic siRNA molecules to knock down the expression of any given disease-associated target gene for therapeutics. SiRNAs are double-stranded RNA duplexes of approximately 21–23 nt in length with 2 nt 3’ overhangs [19,20]. The siRNA triggers are incorporated in the cytoplasm into the RNA-induced silencing complex (RISC), which is a ribonucleoprotein complex, including Dicer, the RNA binding protein TRBP, and Argonaute 2 (AGO2). Upon RISC loading, the double-stranded siRNA is unwound, in which the strand with the less thermodynamically stable 5’-end of the duplex is incorporated and is responsible for guiding the RISC to the complementary target mRNA. The target mRNA cleavage is mediated by the catalytic protein AGO2[19,20], (Fig. 2), and occurs at the phosphodiester linkage between nucleotides 10 and 11 (counting from the 5´-end) between the target mRNA and siRNA guide strand. Once this initial cut is made, the degradation is completed by cellular exonucleases. Moreover, oligouridylation of the newly generated 3′ end of the cleaved RISC products can promote exonucleolytic targeting. The mRNA target dissociates from the intact siRNA after cleavage, freeing RISC to regenerate and cleave additional newly synthesized mRNA targets[21].

Therapeutic approaches that deploy the RNAi pathway require chemical modifications of the siRNA molecules for target mRNA specificity, reduced immune activation, and enhanced stability *in vivo*[11]. Therapeutic siRNAs harbouring 2’ sugar modifications such as 2’-O-Me and 2’-F exhibit reduced immunogenicity and improved serum stability (Fig. 1) [22–25], while substitution of the terminal phosphodiester bonds with PS linkages enhances nuclease resistance of the siRNA molecules[22,26]. Furthermore, incorporation of a 5’(E)-vinyl-phosphonate[27] in the antisense strand has been shown to enhance siRNA potency and duration of action[28], whereas addition of a (S)-glycol nucleic acid modification in the 5’ region of the siRNA sense strand has been reported to enhance potency and lower hepatotoxicity[29,30].

## Delivery of RNA drugs to the CNS

Adequate drug delivery to the brain is one of the biggest hurdles in the development of therapeutics for CNS disorders. Many drug targets have been deemed undruggable due to difficulty encountered with CNS delivery, and thus, new, and improved delivery approaches have become an important aspect of development of RNA-based therapeutics for treatment of neurological and neuromuscular diseases.

**Figure 3.**
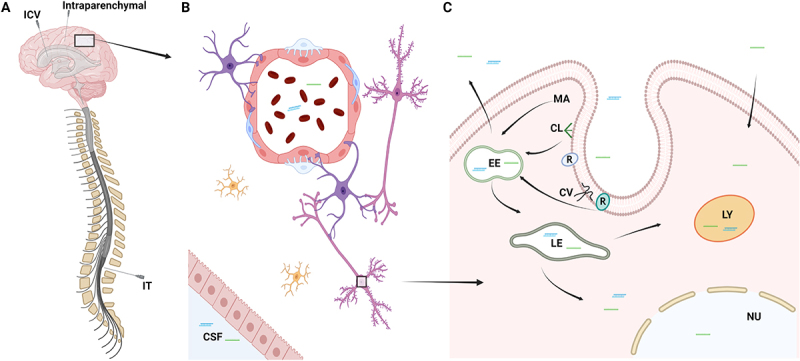
Delivery of RNA therapeutics to the CNS. Schematic illustration depicting the delivery of RNA therapeutics to the CNS. A: Due to the inability to cross the blood-brain barrier, RNA drugs are currently delivered either by intrathecal (IT), intracerebroventricular (ICV), or intraparenchymal injections. B: RNA drugs penetrate and distribute in the brain parenchyma, where they will encounter the different cells of the brain. C: Cellular uptake of RNA drugs can either occur by clathrin- or caveolin-dependent endocytosis, macropinocytosis or other non-productive pathways. CL: Clathrin, CV: Caveolin, EE: Early endosome, LE: Late endosome, LY: lysosome, MA: Macropinocytosis, R: Receptor.

To elicit its function in the CNS, a drug needs to enter the brain parenchyma. The blood-brain barrier (BBB) protects the brain from the periphery and is composed of capillary endothelial cells with tight junctions, which together with astrocytes, pericytes, and neurons comprises the neurovascular unit tightly regulating drug access to the brain[31]. The tight junctions between the endothelial cells render the brain impenetrable to ASOs and siRNAs due to their large size and thus, unassisted uptake of naked RNA drugs through this route is not feasible. Chemically modified ASOs and siRNAs have increased target affinity, improved resistance to nucleases and decreased toxic and immunogenic potential. However, these modifications have not been able to adequately enhance delivery of the RNA drugs across the BBB in the clinic. Thus, the most common strategy to deliver RNA drugs to the brain is to circumvent the BBB by using either intrathecal (IT), intracerebroventricular (ICV) or intraparenchymal administration of the drugs (Fig. 3A). Of these three methods, IT injection in the lumbar region is used in spinal muscular atrophy (SMA) patients receiving nusinersen[32] and is currently the most common administration route for RNA-based therapeutics for neurological diseases and neuromuscular disorders (Table 1). IT injection can either be done by the classic interlaminar injection[33,34], by the transforaminal approach[34,35], but also a subcutaneous (s.c.) catheter pump has been utilized experimentally for repeated injections of nusinersen[36]. Chronically implanted catheters for IT injections have been used in the management of pain and muscle spasticity for decades to deliver e.g. opioids or baclofen directly into the CSF [37,38]. Such devices represent an intriguing prospect to administrate RNA drugs to the CNS, while decreasing the potential psychological stress and physical discomfort associated with repeated IT administrations. For pain management, the chronically implanted pumps report low malfunctioning rates, good tolerability in the patients, and the surgical procedure is well established[38,39]. However, adverse events do occur and include catheter tip granulomas, infections, and hygroma[38]. Lessons from pain and muscle spasticity management show that patient populations need to be carefully selected for the procedure to maximize patient gain and minimize adverse events. IT injection is also used in larger experimental animals such as rats and cynomolgus monkeys[40] to assess the feasibility of IT delivery of RNA drugs during preclinical development as well as establishing dosing schemes and investigate potential adverse effects. In contrast, ICV injection is often carried out in mice[41], but has also been used in humans to deliver e.g. chemotherapy using a port device[42]. Delivery of RNA drugs by this method has not been investigated but represents a potential delivery route if inadequate drug distribution is achieved after IT administration. Intraparenchymal injections in humans are relatively invasive, but AMT-130 (UniQure); a synthetic miRNA delivered by adeno-associated virus-5 in Phase I/II for the treatment of Huntington<apos;>s disease is administered by intrastriatal injections[43]. Delivery of RNA therapeutics to the brain by intranasal administration allowing the RNA drugs to travel to the CNS via the olfactory nerves has also been investigated. This route of administration remains at the experimental level due to challenges with bioavailability and lack of adequate and correct dosing[44]. Another method for enhancing brain delivery of RNA drugs is short-term BBB disruption, where the BBB is transiently opened by focused ultrasound[45,46], hyperosmolar agents[47], disease pathology[48,49], or a tight junction modulator[50] to facilitate transfer of the RNA drug to the brain parenchyma. While intriguing at an experimental level, the applicability of this approach to the clinical setting remains uncertain due to lack of dosing control, potential adverse side effects, and the risk of co-introducing unwanted components to the brain. The reversibility of the disruption also needs to be investigated thoroughly.

**Table 1.** RNA therapeutics in clinical development for treatment of neurological and neuromuscular diseases

Therapeutic name	Condition(s)	Target(s)	Chemical modification	Delivery system	Sponsor company	Clinical phase	Status	NCT number
**Neuromuscular disease**								
Spinraza (Nusinersen)	Spinal muscular atrophy (SMA)	SMN-2 exon 7	18mer PS 2’‐MOE (splice modulator ASO)	IT	Ionis Pharma, Biogen	III, approved	Commercialized	NCT02193074 NCT02292537
Exondys 51 (Eteplirsen)	Duchenne muscular dystrophy (DMD)	Dystrophin exon 51	30mer PMO (steric block ASO)	IV	Sarepta Therapeutics	III, approved	Commercialized	NCT02255552
Vyondys 53 (Golodirsen)	Duchenne muscular dystrophy (DMD)	Dystrophin exon 53	25mer PMO (steric block ASO)	IV	Sarepta Therapeutics	III, approved	Commercialized	NCT02500381
Amondys 45 (Casimersen)	Duchenne muscular dystrophy (DMD)	Dystrophin exon 45	22mer PMO (steric block ASO)	IV	Sarepta Therapeutics	III, approved	Commercialized	NCT02500381
Viltepso (Viltolarsen)	Duchenne muscular dystrophy (DMD)	Dystrophin exon 53	ASO PMO	IV	Nippon Shinyaku	III, approved	Commercialized	NCT04337112
**Polyneuropathies**								
Onpattro (Patisiran, ALN‐TTR02)	Familial amyloid polyneuropathy (FAP)	TTR	siRNA (2’‐OMe, 2’‐F)	IV	Alnylam Pharmaceuticals	III, approved	Commercialized	NCT02939820
Tegsedi (Inotersen)	Familial amyloid polyneuropathy (FAP)	TTR	ASO, 2’-MOE, gapmer	SC	Ionis Pharma /Akcea Therapeutics	III, approved	Commercialized	NCT01737398
**Neuromuscular disease**								
SRP-5051	Duchenne muscular dystrophy (DMD)	Dystrophin exon 51	PPMO ASO	IV	Sarepta Therapeutics	I	Completed	NCT03375255
Tofersen (BIIB067)	Amyotrophic Lateral Sclerosis (ALS)	SOD1	ASO	IT	Ionis Pharma/Biogen	III	Completed	NCT02623699
Jacifusen (ION363)	Amyotrophic Lateral Sclerosis (ALS)	FUS	ASO	IT	Ionis Pharma	III	Recruiting	NCT04768972
BIIB078-IONIS-C9_Rx_	Amyotrophic Lateral Sclerosis (ALS)	C9ORF72	ASO	IT	Ionis Pharma/Biogen	II	Discontinued	NCT03626012
BIIB100	Amyotrophic Lateral Sclerosis (ALS)	XPO1	ASO	IT	Biogen	I	Completed	NCT03945279
IONIS-DNM2-2.5_Rx_	Centronuclear Myopathy (XL-CNM, AD-CNM)	DNM2	ASO	IV	Ionis Pharma /Dynacure	II	Recruiting	NCT04033159
**Neurological disease**								
BIIB080	Alzheimer's Disease (AD)	MAPTR	ASO	IT	Ionis Pharma /Biogen	I/II	Active, not recruiting	NCT03186989
BIIB094 (ION859)	Parkinson's Disease (PD)	LRRK2	ASO	IT	Ionis Pharma /Biogen	II	Recruiting	NCT03976349
BIIB101 (ION464)	Parkinson's Disease (PD)/Multiple System Atrophy (SMA)	SNCA	ASO	IT	Ionis Pharma /Biogen	II	Active, not recruiting	NCT04165486
ALT1102	Multiple Sclerosis (MS)	CD49d	ASO	SC	Antisense Therapeutics Limited	II	Completed	ACTRN12608000226303
**Polyneuropathies**								
Vutrisiran (ALN‐TTRsc02)	Familial amyloid polyneuropathy (FAP)	TTR	siRNA (PS, 2’‐OMe, 2’‐F)	SC	Alnylam Pharmaceuticals	III	Active, not recruiting	NCT03759379
Eplontersen (AKCEA-TTR-LRx)	Familial amyloid polyneuropathy (FAP)	TTR	ASO	SC	Ionis Pharma/ Akcea Therapeutics	III	Recruiting	NCT04136184
**Repeat-diseases**								
Tominersen	Huntington's diseases (HD)	HTT	ASO	IT	Ionis Pharma/Roche	III	Terminated	NCT03761849
WVE-120101	Huntington's diseases (HD)	HTT	ASO	IT	Wave Life Sciences/Takeda	I/II	Terminated	NCT03225833
WVE-120102	Huntington's diseases (HD)	HTT	ASO	IT	Wave Life Sciences/Takeda	I/II	Terminated	NCT03225846
AMT-130	Huntington's diseases (HD)	HTT	miRNA/AAV5	IST	UniQure	I/II	Recruiting	NCT04120493
**Others**								
RO7248824	Angelman syndrome (AS)	UBE3	ASO	IT	Roche/Genentech	I	Recruiting	NCT04428281
GTX-102	Angelman syndrome (AS)	UBE3	ASO	IT	GeneTX Biotherapeutics/ Ultragenyx Pharmaceutical	I/II	Active, not recruiting	NCT04259281
ION373	Alexander disease (AxD)	GFAP	ASO	IT	Ionis Pharma	III	Recruiting	NCT04849741
STK-001	Dravet syndrome	SCN1A	ASO	IT	Stoke Therapeutics	II	Enrollment by invitation	NCT04740476

Once the drug has penetrated the parenchyma, the level of drug distribution within the brain is crucial for its therapeutic effects (Fig. 3B). Many neurological disorders such as Alzheimer's disease and Parkinson's disease affect specific neuronal/glial cell populations in selective brain areas. Thus, effective treatment requires that the drug reaches small well-defined cell populations in the brain, e.g. the midbrain dopaminergic neurons of the *substantia nigra* and *ventral tegmental area* in Parkinson's disease[51]. These neurons comprise approximately 600,000 cells in the adult human brain, whereas the entire human brain consists of ca. 80 billion neurons and almost the same number of glial cells[52] highlighting the importance of adequate drug distribution.

The final step for the drug to elicit its therapeutic effect is cellular uptake (Fig. 3C). ASOs have been proposed to be taken up by clathrin or caveolin-dependent endocytosis after interaction with different receptors and proteins at the cell surface, while encapsulated siRNAs are primarily taken up by macropinocytosis[10]. Intracellular trafficking of ASOs and siRNAs is still poorly understood, however, endosomal escape of RNA drugs is low[53]. A higher rate of endosomal escape of an RNA drug requires drug uptake by the individual cell to be proportionally larger for the drug to elicit a therapeutic effect. This underscores assessment of cellular delivery in development of RNA therapeutics to ensure adequate efficacy of the drug[54].

Despite the ability to bypass the BBB by IT or ICV injections, cellular uptake, and adequate distribution of RNA drugs in the CNS remain challenging. Thus, development of different carrier systems to enhance BBB transport, increase CNS distribution, and cellular drug uptake has become an active field of research aimed at improving CNS delivery of RNA drugs. Bioconjugation of RNA molecules has shown promising results upon the development of N-acetylgalactosamine (GalNac)-conjugated RNA therapeutics for delivering the RNA cargo to hepatocytes in the liver[55,56]. RNA drugs can be conjugated to different chemical moieties such as peptides, antibodies, lipids, or sugars, to promote targeted delivery, bioavailability, and cellular uptake, and such bioconjugates have also been tested for delivering RNA-based therapeutics across the BBB. Cholesterol has been utilized in both ASOs and siRNAs to enhance delivery and distribution in the CNS by increasing their association with plasma membranes and internalization of the bound RNA drugs[41,57,58]. However, a drawback of cholesterol is its limited CNS distribution[58]. The transferrin receptor has also been broadly utilized as a delivery strategy to target CNS drugs to the brain via conjugation of the cargos to anti-transferrin receptor antibodies[59,60]. The receptor is exclusively expressed at the luminal site of endothelial cells in the brain capillaries and conveys transfer of iron into the brain, and thus it can function as a drug shuttle into the CNS. However, systemic delivery of an siRNA conjugated to an anti-transferrin receptor antibody showed very low CNS bioavailability[60], and further research is therefore required to optimize antibody-mediated delivery of RNA drugs to the brain. Cell-penetrating peptides (CPPs) comprise another promising class of bioconjugates for enhanced CNS delivery. CPPs are short (< 30 amino acids), amphiphilic, or hydrophilic peptides with the ability to translocate to the cytoplasm either directly through the membrane or through endocytosis[8,61]. One such CPP is PMO internalizing peptide (Pip)6a. Conjugation of Pip6a to a splice modulator PMO targeting exon 7 in *SMN2* facilitated delivery of this compound into the brain and spinal cord upon systemic delivery and resulted in increased tissue levels of the full-length survival motor neuron protein. Furthermore, treatment with the Pip6a-conjugated PMO rescued the SMA phenotype and extended the life span in a mouse model of SMA[62].

CPPs can also be conjugated with other peptides to enable cell specific delivery. For example, a nona (D-arginine) peptide was fused to the rabies virus glycoprotein, which binds the acetylcholine receptor present in neurons. The resulting RVG-9 R CPP was shown to bind and deliver an siRNA cargo to Neuro 2a cells *in vitro*. Moreover intravenously administered RVG-9 R-siRNA showed enhanced delivery of the compound across the BBB in mice and silencing of superoxide dismutase 1 (SOD1) in the mouse brain [63,64].

Additional delivery methods include the use of nanotechnology to package RNA drugs into protective structures such as exosomes, spherical nucleic acids (SNAs), DNA nanostructures, and lipid nanoparticles (LNPs). Exosomes are naturally occurring extracellularly secreted lipid bilayer vesicles with a diameter of 40 to 150 nm and carry out important functions in intercellular communication[65]. They contain different cellular components including mRNAs and noncoding RNAs that are released into recipient cells by plasma membrane fusion, endocytosis, or phagocytosis, as well as other routes[66]. Due to the lipid bilayer composition, exosomes readily cross biological membranes, including the BBB making them interesting as carriers for RNA cargos into the CNS. Targeting of exosomes to the brain is feasible by incorporating CNS-specific delivery components in the lipid bilayer such as the rabies virus glycoprotein peptide[67–69]. Exosomes have been proposed to elicit anti-inflammatory functions and thus could potentially provide additional therapeutic advantages in CNS diseases with pro-inflammatory pathogenesis in conjunction with the therapeutic cargo[70]. Furthermore, exosomes are non-toxic and non-immunogenic[71], have the potential for carrying multiple RNA drugs simultaneously, and can facilitate RNA drug delivery to the CNS via intravenous administration[67,68]. Among the disadvantages in using exosomes for CNS delivery are the cargo loading procedures. These can be divided into passive such as incubation with donor cells or isolated exosomes[72] or active such as electroporation sonication, extrusion, and use of membrane permeabilizers[67–69], all of which have distinct disadvantages in terms of efficiency and technical difficulties[72].

Lipid nanoparticles (LNPs) consist of lipid bilayers that encapsulate the RNA drug cargo, thereby protecting it from nucleases and endosomal breakdown. The lipids can either be solid or liquid-crystalline or a combination hereof and are manufactured in well-defined combinations with surfactants and other components, including targeting ligands to increase their pharmacokinetic properties[73]. Due to their large size, LNPs do not cross the BBB on their own, but ICV injected LNPs have shown widespread distribution and gene knockdown in the CNS[74,75]. A drawback of LNPs is their potential toxicity and immunogenicity[76]. For example, patisiran, an LNP-formulated siRNA drug for hereditary transthyretin amyloidosis requires pretreatment with steroids and antihistamines due to adverse reactions[77].

SNAs are formed by specifically oriented siRNA or ASOs attached in a high-density to a hydrophobic core composed of e.g. gold or liposomes by a linker and therefore the RNA molecules are exposed to their surroundings[78]. SNAs are resistant to nucleases, can cross the BBB, and are readily taken up by cells through scavenger receptors[78]. SNAs have primarily been used for treatment of glioblastoma multiforme due to the high accumulation in these tumors probably due to disease-induced compromised BBB facilitating high local uptake [49,79]. Whether SNAs are capable of crossing the BBB in neurological diseases lacking a compromised BBB remains to be investigated.

DNA nanostructures such as DNA cages are self-assembled into precise structures due to base-pairing and can be designed for optimal pharmacokinetic properties and loading potential including loading of RNA drugs[80]. The challenges of DNA nanostructures include low retention times due to degradation, poor cellular delivery due to endosomal breakdown, and preferential uptake into hepatic and renal tissues, however, BBB crossing of DNA cages has been reported[81].

Despite the technological advances in enhanced RNA drug delivery to the CNS, this area of research needs further maturation and scientific advances before the search for a ‘brain GalNac’ is completed. Once the challenge of CNS delivery of systemically administered RNA therapeutics is solved, many patients suffering from intractable neurological disorders will see new therapies becoming available.

## Clinical development of RNA medicines for treatment of neurological and neuromuscular diseases

The RNA drug, Spinraza, was approved in 2016 by the FDA for treatment of the neuromuscular disease SMA[82]. Subsequently, many biopharmaceutical companies have established extensive RNA drug pipelines focusing on treatment of CNS disorders. There are currently 27 RNA-based therapeutics in clinical trials and seven drugs that are commercialized for treating neurological and neuromuscular diseases such as SMA, Duchenne muscular dystrophy (DMD), and familial amyloid polyneuropathy (FAP) (Table 1). These target gene mutations cause a partial or complete loss of function (LoF), gain of function (GoF), or a combination of both. If the disease mechanism is linked to a LoF mutation, the therapeutic strategy aims at restoring the expression of the affected protein product (‘gene replacement’). The goal of a GoF mutation is to reduce the expression of the protein product (‘gene silencing’). Currently, five biopharmaceutical companies, Ionis Pharmaceuticals, Biogen, Roche, Akcea Therapeutics, and Alnylam Pharmaceuticals, respectively, have RNA drugs in phase III trials for treating neurological or neuromuscular diseases. Ionis, Biogen, Dynacure, and Roche have ten ASO-based drugs in clinical trials, including five ASO-based drugs in phase III studies for the treatment of amyotrophic lateral sclerosis (ALS), FAP, Alexander disease (AxD), and Huntington's disease (HD) (Table 1).

ALS is a rare neurological disorder classified as a motor neuron disease that involves the loss of nerve cells located in the spinal cord responsible for controlling voluntary muscle movement[83]. ALS often develops sporadically and progressively, and most commonly affects men between the ages of 55 and 75. As the disease progresses, the patients experience difficulty walking, breathing, speaking or forming words, or dysphagia (swallowing). Patients with ALS often become anxious and depressed because their higher mental processes such as understanding, remembering, reasoning, and problem solving are intact, and thus, the patients are aware of the progressive decline in motor function[83]. The prevalence of ALS is about one in 20,000 and 5–10% of all ALS cases are familial (named as FALS), and most often caused by autosomal dominant mutations. About 25–40% of FALS cases and a small percentage of sporadic cases are caused by an intronic hexanucleotide repeat expansion in the chromosome 9 open reading frame 72 (C9ORF72)-encoding gene, which is expressed in motor neurons and nerve cells in the brain. Another 12–20% of FALS results from mutations in the SOD1 gene, whereas a smaller number of FALS patients have a mutation in the fused-in-sarcoma (FUS) gene[83]. The pharmaceutical company Ionis has developed ASOs against all three targets: BIIB078 for C9ORF72 (phase II trial discontinued as of March, 2022, see Table 1 for more information[84]), jacifusen for FUS, and tofersen for SOD1 (co-sponsored by Biogen)[85]. Jacifusen is an ASO targeting the mutated neurotoxic form of the FUS protein, P525L, which is responsible for an aggressive and rapidly fatal form of ALS that begins in childhood or early adulthood. Last year, Ionis initiated a phase III trial with 64 patients who received either jacifusen or placebo administered IT starting every four weeks, and then every eight weeks, for 29 weeks, followed by a 72-week open-label extension[85].

Tofersen is an investigational ASO targeting the gene encoding SOD1 and is currently in a phase III trial[84]. Results from a 28-week initial study with 108 enrolled patients living with SOD1-ALS receiving either tofersen (*n* = 72, 100 mg tofersen) or placebo (*n* = 36) failed to meet the primary endpoint of change in the Revised Amyotrophic Lateral Sclerosis Functional Rating Scale (ALSFRS-R), which is a validated rating instrument for monitoring progression of disability in patients with ALS[84]. However, the study also reported positive results on multiple other factors, such as reduction of the SOD1 protein in the CSF and reduction of neurofilament light chain, a biomarker of neurodegeneration. Moreover, the study showed signs of slowing disease progression in patients with SOD1-ALS, and an early initiation of tofersen treatment led to slower decline across multiple measures such as motor function, respiratory function, muscle strength, and quality of life[84].

Familial amyloidosis is caused by GoF mutations in the *ATTR* gene leading to a misfolded, liver produced aggregation-prone transthyretin (TTR) protein that is deposited in amyloid aggregates. Amyloidosis causes cardiomyopathy and/or polyneuropathy (PNP) and under these circumstances the disease is called FAP[86]. Before deploying RNA-based therapeutics, treatment of FAP was based on liver transplantation or ineffective TTR stabilizers such as tafamidis or diflunisal. Two RNA-based therapies, vutrisiran and eplontersen, have been developed for the treatment of PNP caused by familial *ATTR* amyloidosis. Eplontersen is developed by Ionis /Akcea Therapeutics and is administered s.c. once every four weeks. The GalNAc-conjugated ASO utilizes the tissue ligation system, named LICA, to specifically target the liver. Vutrisiran is a GalNAc-conjugated siRNA-drug administered s.c. once every three months and was developed by Alnylam Pharmaceuticals[87]. Conjugating the siRNA with a GalNac moiety allows for selective delivery of the RNA drug to hepatocytes in the liver[88].

The aforementioned RNA drugs promote degradation of the mutant and the wildtype *ATTR* mRNA through RNAse H (eplontersen) and RISC (vutrisiran), respectively. Eplontersen has recently entered a clinical phase III study and completed the enrollment of 160 patients with hereditary transthyretin-mediated amyloid polyneuropathy[89]. Results from the phase I trial showed a reduction in TTR of up to 94% in patients treated with eplontersen[89]. Vutrisiran is based on the same siRNA sequence as the already approved patisiran but utilizes the GalNAc delivery platform instead of LNPs. HELIOS-A Phase III study of vutrisiran showed good safety, tolerability, and efficacy with a total dose of 25 mg every three months for nine months. Up to 83% reduction of TTR after a single 25 mg dose of vutrisiran was observed, and there were no drug-related discontinuations or deaths after nine months[87]. Currently, there are two RNA drugs approved and commercialized for the treatment of TTR, patisiran and inotersen developed by Alnylam and Ionis, respectively[88].

HD is a rare, inherited, genetic progressive disease that is caused by the expansion of CAG trinucleotide repeats in the huntingtin (HTT) gene[90]. The mutation in the HTT gene is toxic and leads to gradual destruction of neurons causing mental and physical disabilities as the disease progresses. The first symptoms often appear as movement, cognitive, and psychiatric disorders with onset at the age of 30 to 40 years. If the disease develops early, it is classified as juvenile HD. Presently, there are no cures or effective treatments for HD and most patients die of pneumonia, injuries from falling, or complications related to inability to swallow within 10–30 years after disease initiation. Juvenile HD usually results in death within 10 years after the first symptoms develop[90]. Tominersen, initially developed by Ionis and subsequently licensed to Roche, is an investigational ASO targeting the HTT-encoding gene[91]. Tominersen is administered by IT injections using a dose of 120 mg every second or fourth week. Results from the phase I/II trial showed that tominersen significantly reduced the level of HTT protein in the CSF and had a good safety, tolerability, pharmacokinetic, and pharmacodynamic profile in patients receiving either the high (120 mg) or low (30 mg) dose of the ASO drug. These results facilitated a phase III study with 791 HD patients, the largest clinical trial in HD to date. The study participants were divided into three groups, a placebo group, a group receiving tominersen every eight weeks, and a second group receiving tominersen every 16 weeks[91]. In March 2021, Roche announced that dosing of tominersen should be halted based on minimal observed benefits in the patients[92]. Results showed that the more frequent dosing was associated with more severe adverse events such as hydrocephalus (excess CSF buildup in the brain), headache, motor problems, and infections. The 16-week dosing group had a similar score on measures of functional ability, motor function, and cognition compared to placebo. Roche continue to follow the study participants for safety and efficacy measures and tominersen is still in a phase III clinical trial without any further dosing planned [92,93].

## Other neurological diseases in clinical trials

### Alexander disease

Alexander disease (AxD) is a rare, fatal, and progressive neurological condition characterized as a leukodystrophy (abnormal growth of white matter in the brain)[94]. Four major types of AxD have been defined. The neonatal form leads to severe disability or death within the first two years. Symptoms in the infantile onset form include abnormal head size, seizures, limb stiffness, delayed or declining cognition development, and lack of growth. The juvenile type with disease onset after the age of four years presents with difficulties in speaking and swallowing, excessive vomiting, poor coordination, and loss of motor control. The rare adult type AxD shows symptoms similar to Parkinson's disease and multiple sclerosis such as impairment of speech, muscle weakness, difficulty walking, and uncontrolled spasms depending on the affected area. Approximately 90% of AxD cases are caused by an autosomal dominant GoF mutation in the gene glial fibrillary acidic protein (GFAP) leading to overproduction and accumulation of GFAP with formations of Rosenthal fibers in astrocytes. Currently, there are no approved treatments available, and patients only receive symptomatic and palliative care[94]. ION373 is an investigational ASO targeting the *GFAP* mRNA for the treatment of AxD and is currently in phase III trials. This study is carried out as a double-blinded placebo-controlled study with 58 patients with AxD that will receive an IT injection once every 12 weeks through week 109. Results from this clinical study are expected in 2024[95].

### Angelman syndrome

Angelman syndrome (AS) is a rare genetic neurological disorder caused by loss of the UBE3A protein due to a *de novo* deletion of the maternally inherited 15q11-q13 region encompassing the *UBE3A* gene[96]. The paternal copy of UBE3A is known to be imprinted within the hippocampus, cortex, thalamus, olfactory bulb, and cerebellum due to silencing by the *UBE3A* antisense transcript (UBE3A-AS). Thus, a functional maternal copy is essential for proper development. AS affects 1 in 12,000 to 20,000 people with equal frequency between males and females. The first symptoms become noticeable by the age 6–12 months, and include intellectual disability, impaired motor coordination, seizures, abnormal sleep, lack of speech, and high comorbidity with autism spectrum disorders[96]. Two ASO-based drugs targeting the parental *UBE3A*-antisense transcript to reactivate the paternal UBE3A allele in neurons are currently in clinical studies by Roche/Genetech and Gene Biotherapeutics/Ultragenyx Pharmaceutical, respectively[97,98].

### Centronuclear myopathies

Centronuclear myopathies (CNM) comprise a group of rare genetic congenital muscle disorders characterized by abnormal localization of the nucleus in the center of skeletal muscles cells. Patients with CNM experience muscle weakness slowly worsening over time, breathing problems, cardiomyopathy, neuropathy, and intellectual disability. Lung failure or infection can lead to early infancy death. Mutations in several genes have been identified for CNM. The most severe form is the X-linked CNM (XL-CNM), which is caused by LoF mutations in the myotubularin *MTM1* gene, while three different genes, DMN2, BIN1, and RYR1, have been identified to cause an autosomal dominant CNM (AD-CNM) form[99]. Ionis has an investigational ASO (IONIS-DMN2-2.5_rx_) in a phase I/II trial for the treatment of DNM2 AD-CNM, which accounts for 50% of all CNM patients. IONIS-DMN2-2.5_rx_ is an ASO targeting the gene encoding dynamin 2 (DMN2), which leads to abnormalities primarily in the distal lower leg muscle[100].

### Alzheimer's disease

Alzheimer's disease (AD) is a progressive neurological disorder that leads to neuronal death and shrinking of the brain parenchyma[101]. AD is the most common cause of dementia, and shows symptoms such as cognitive, behavioral, and social decline that affect the patients’ ability to perform daily activities. Approximately 50 million people worldwide suffer from dementia, and between 60–70% of these are diagnosed with AD. The exact pathogenesis of AD is unknown, but two pathological proteins; β-amyloid and tau have been linked to AD. The β-amyloid protein is a fragment of a larger protein that disrupts cell-cell communication when clustered as amyloid plaques, whereas the tau protein is important for transporting nutrients in the brain. However in AD, the transport system is disrupted causing cell death due to changes in the shape of the tau protein, leading to structures called neurofibrillary tangles[101]. Ionis has developed an investigational ASO (BIIB080) against the gene encoding microtubule-associated protein tau (MAPT) in the brain. The ASO is administered monthly by IT injections and has demonstrated dose-dependent reduction in total tau and phosphor-tau proteins in the CSF by 30–50% in clinical phase I/II trials, while no serious adverse events were reported[102].

### Parkinson's disease

Parkinson's disease (PD) is a progressive neurodegenerative disease characterized by loss of dopaminergic neurons in *substantia nigra* of the motor system. PD is the second most common neurodegenerative disease affecting about 1% of the population over age 60. Patients present with tremors, loss of balance and coordination, rigid muscles, slowing of movement, changes in speech and sense of smell, sleep disorder, and in some cases cognitive decline. PD can be idiopathic and familial, where hereditary mutations include a GoF mutation in the leucine-rich repeat kinase 2 (LRRK2) gene and dominant mutations in the SNCA gene. Mutation in the LRRK2 is the most common genetic mutation in PD, and induces neuronal cell death in tissue culture, but the mechanism in humans remains uncertain. However, there are no differences in clinical features between hereditary PD and idiopathic PD, suggesting that LRRK2 is implicated in all forms of PD[103]. ION859 (also known as BIIB094) developed by Ionis, is an ASO targeting the LRRK2 gene, and thereby suppresses production of the LRRK2 protein. The ASO is currently in phase I trial evaluating safety and tolerability of single and multiple doses administered IT to study subjects with PD. The first study data are expected in 2022[104].

### Multiple system atrophy

Multiple system atrophy (MSA, also called Shy-Drager syndrome) is a rare, rapidly progressing, fatal neurodegenerative disease affecting the autonomic functions such as blood pressure, breath, bladder function, and motor function. Symptoms of MSA often appears between age 50–60 but can begin as early as age 30. Two types of MSA are classified: the parkinsonian and the cerebellar type. The former shares many symptoms with PD, whereas the latter presents with ataxia, autonomic changes, dysarthria, and vision disturbances. Accumulation of the α-synuclein protein in MSA is unique in that it is prominent not only in neurons, but also in glia cells[105]. Ionis has developed an ASO drug named ION464 (also known as BIIB101) targeting the alpha-synuclein gene, SNCA, for the reduction of the levels of alpha-synuclein in the brain. Aberrant accumulation of alpha-synuclein in the brain is thought to be a driver of the pathogenesis in both PD and MSA. The ASO is currently in a phase I trial and is evaluated for safety and tolerability of multiple doses administered via IT injections to patients with MSA. The first study data are expected in July 2022[106].

### Multiple sclerosis

Multiple sclerosis (MS) is a devastating neurological disease that progressively destroys the insulating myelin sheaths surrounding neurons causing symptoms such as blurred vision, difficulty walking, numbness, and ultimately paralysis and death. The risk factors include female gender, sun exposure and vitamin D, Caucasian ethnicity, smoking, and obesity. The onset of MS is usually between age 20–30 but can occur at any age. Since progression of MS involves infiltration of T-cells and B-cells from the periphery into the CNS, most MS therapies focus on reducing systemic inflammation[107]. The VLA-4 receptor is important for immune cell adhesion and consists of two subunits, CD49d and CD29, where especially CD49d positively correlates with the severity of MS. Antisense Therapeutics has developed an ASO (ALT1102), which is currently in a phase II trial for treatment of patients with relapsing-remitting multiple sclerosis (RRMS) and DMD. This ASO targets the CD49d receptor and has reported highly promising results in reducing the number of brain lesions in MS patients[108].

### Dravet syndrome

Dravet syndrome (also known as severe myoclonic epilepsy of infancy) is a rare, genetic epileptic encephalopathy characterized by episodes of prolonged seizures beginning in infancy or early childhood. The prevalence of Dravet syndrome is 1 in 20,000 to 40,000 and in 80–90% of Dravet patients, the disease is caused by *de novo* mutations in the SCN1A gene, a sodium channel required for proper function of neurons. The severity of the disease is correlated with the location and type of the mutation. Most children experience intellectual disability, ataxia, autistic behavior, and sleep disorders, and do not respond well to conventional anti-epileptic treatment such as clobazam and valproic acid[109]. Stoke Therapeutics has developed an ASO, named STK-001, targeting the SCN1A gene. It is currently in phase II trials, where the safety and tolerability of this drug after multiple IT administered doses of up to 70 mg will be evaluated. The first interim data from 21 patients showed that STK-001 is well-tolerated with no safety concerns, and reduced seizure frequency after a single injection[110]. Stoke is currently recruiting more patients for phase I trial, which is estimated to complete in March 2024[111].

## Approved RNA drugs for neurological diseases

**Figure 4.**
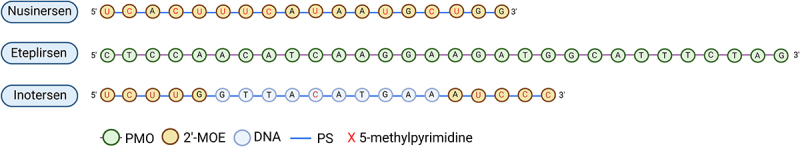
Chemical compositions of nusinersen, eteplirsen, and inotersen. Nusinersen is an 18-nucleotide fully phosphorothioate (PS)-modified 2’-*O*-methoxyethyl (2’-MOE) ASO targeting survival motor neuron 2 (SMN2) for the treatment of spinal muscular atrophy (SMA). Eteplirsen is a 30-nucleotide PMO-based drug targeting exon 51 of the Dystrophin gene (DMD). Inotersen is a 20-mer gapmer ASO targeting transthyretin (TTR). The ASO is fully PS-modified with five 2ʹ-MOE-modified ribonucleotides at each terminus (i.e. 5–10-5 structure).

There are currently seven FDA-approved RNA-based drugs on the market for the treatment of neurological and neuromuscular diseases. Nusinersen (Spinraza) was approved by the FDA for treatment of SMA in 2016, whereas four drugs have been approved for DMD: eteplirsen (Exondys51), golodirsen (Vyondys53), casimersen (Amondys53), and viltolarsen (Viltepso), and two for the treatment of FAP: patisiran (Onpattro) and inotersen (Tegsedi) (Table 1). In this section, we will describe three of the aforementioned RNA drugs in more detail (Fig. 4).

SMA is a severe autosomal recessive and progressive disease affecting both the CNS and peripheral nervous system (PNS), and thereby also affecting voluntary muscle movement. SMA is classified as a motor neuron disease that involves the loss of the neurons in the spinal cord responsible for the innervation of muscles. The lack of innervation causes muscular atrophy, which can result in death due to failure of respiratory muscles and pneumonia[82]. SMA affects 1 in 11.000 children and is the most common genetic cause of mortality in infants. In >95% of patients, SMA is caused by homozygous LoF mutations in the gene encoding the survival motor neuron 1 protein (SMN1). The disease is divided into four subtypes (1–4) based on disease severity and age of onset, where SMA type 1 is the most severe form. The severity often depends on the copy number of the homologous SMN2 gene, which due to alternative splicing does not have exon 7, and as a result, not a fully functional survival motor neuron protein. The approved SMA drug Spinraza introduces exon 7 in SMN2 by altering splicing of the pre-SMN2 resulting in functional full-length SMN protein in SMA patients[82]. Spinraza is an 18-mer 2’-MOE-modified ASO drug and the first RNA therapy approved for the treatment of SMA[82]. Phase I/II trials of Spinraza examined the safety, pharmacokinetic, and efficacy profile, and proved the drug to be well-tolerated across all studied age groups. The studies showed improvement in the motor functions across SMA of all types [112,113]. Spinraza is administered IT every 4 months after four initial doses, and significantly improves motor function, and the ability to breathe and swallow. Adverse events like upper airway infections, headache, constipation, back pain, and post–lumbar puncture syndrome have been reported[82].

Muscular dystrophies (MD) are a group of more than 30 different inherited genetic diseases that gradually cause progressive weakness and loss of muscle mass, leading to progression of physical disability[114]. There are various types of MD, each presenting with different symptoms, degree of severity, and impact on the everyday life. The most common types of MD are Duchenne MD (DMD), Becker MD (BMD), limb-girdle MD (LGMD), myotonic dystrophy, facioscapulohumeral MD, Pompe disease, oculopharyngeal MD, and Emery-Dreifuss MD. DMD is an X-linked recessive genetic myopathy caused by mutations in the gene dystrophin *(DMD)*. Lack of dystrophin protein in muscle cells leads to breakdown of muscles. Depending on the mutation, it can lead to either a more severe phenotype of DMD (complete LoF) or a milder form of BMD (partial LoF)[114]. The prevalence of DMD is approximately 6 in 100,000 individuals and it primarily affects boys. DMD begins around the age of two years, and affects first proximal muscles, then distal limb muscles, and later the heart and respiratory muscles. Until recently, individuals with DMD did not survive beyond their teen years, but with medical treatment, and cardiac and respiratory care, the life expectancy is increasing [114,115]. There are currently nine RNA-based drugs in clinical trials for the treatment of DMD, LGMD, and Pompe disease, including four approved drugs for DMD (see Table 1). Eteplirsen (Exondys51), golodirsen (Vyondys53), casimersen (Amondys53), and viltolarsen (Viltepso) have been approved for the treatment of DMD in 2016, 2019, 2020, and 2021, respectively, by the FDA[116]. All drugs are designed to target different exons of the dystrophin gene.

Eteplirsen (Exondys51) is a single-stranded 30-nucleotide neutrally charged phosphorodiamidate morpholino oligomer (PMO). Eteplirsen targets exon 51 of DMD causing an exclusion of this exon during pre-mRNA processing. By skipping exon 51, the translational reading frame is corrected resulting in the production of a functional, but truncated dystrophin protein. Approximately 20% of DMD patients (with *DMD* mutations) have a deletion at the end of exon 50 and the beginning of exon 52 and will thus benefit from eteplirsen treatment. Eteplirsen is the first FDA-approved RNA drug for treatment of DMD[117]. However, the efficacy of eteplirsen has been questioned. Although the patients treated with 30 mg/kg/week IV had a significant (51.7% after 48 weeks) increase of dystrophin-positive fibers compared to the placebo, the clinical benefit (based on a 6-minute walk test) was unchanged. On the other hand, eteplirsen was shown to affect pulmonary function positively in patients[117]. The lack of efficacy has been ascribed to the PMO chemistry, since PMOs exhibit rapid clearances and poor tissue uptake[118]. However, in addition to safety, PMOs may exhibit additional advantages. Due to their neutral charge, PMOs do not interact with proteins such as nucleases, which makes them highly stable[16]. In clinical trials eteplirsen was shown to be well-tolerated with no observed adverse events, and neither hepatic nor renal functions were compromised, while serum chemistries were within normal ranges, and no immunogenic responses were observed. At present, eteplirsen is far from curative and shows only a marginal effect on improving DMD clinical manifestations highlighting the need for improved therapeutics[117].

Inotersen (Tegsedi) is an ASO targeting the gene encoding TTR for the treatment of FAP[119]. As the disease progresses, patients experience increasing lower limb muscle weakness, walking difficulty, imbalance, and sensory loss because of injury to the somatic and autonomic nerve fibers. The degree of neurological manifestations of FAP may vary depending on the mutation type (more than 140 TTR genetic mutations are identified), however, these patients experience a substantial burden on their health-related quality of life (HRQOL)[120]. Inotersen is a 20-mer fully-PS chimeric gapmer ASO with five 2’-MOE-modified sugars at each terminus (i.e. 5–10-5 structure), showing a knockdown efficiency of 80% in a human TTR Ile84Ser transgenic mouse model. Inotersen reduced levels of both the mutated and wild-type TTR in humans and showed no off-target effects. A phase I clinical trial included 65 healthy volunteers, and four doses of 300 mg s.c. of inotersen resulted in a TTR reduction of 75% in serum[121]. Subsequently, a 300 mg s.c. once weekly inotersen dose was tested in a phase III clinical trial, which included 172 patients (112 patients received inotersen and 60 patients placebo)[119]. Both the primary endpoint; improvement in neuropathy impairment, and secondary endpoints, including a 10-m walk test were met. Common adverse events included injection site reactions, nausea, headache, fever, and vomiting, but also thrombocytopenia was apparent presenting a more severe side effect, and therefore close motoring of blood platelet counts during treatment with inotersen is necessary[119,120]. Interim data on the long-term effect of up to three years of inotersen preserved HRQOL, however a longer lasting compound based on the same therapeutic principle (AKCEA-TTR-LRx, eplontersen) is currently being tested in phase III trials[122]. Although mRNA-specific therapies effectively prevent newly formed amyloids by suppressing the production of plasma TTR, these treatments do not remove amyloid from tissues and organs. These findings underscore the importance of early treatment for patients with familial amyloidosis.

## Concluding remarks

RNA-based therapeutic approaches hold great promise in the treatment of a wide variety of human diseases, including CNS disorders. Therapeutic ASOs and siRNAs represent the most clinically advanced RNA drug platforms and exhibit several advantages compared to NCE- and NBE-based drugs such as efficient targeting of previously undruggable targets, the potential to modulate entire disease pathways, rapid screening of lead candidates and readily scalable drug manufacturing process. Furthermore, advances in chemical modifications have enhanced the drug-like properties of ASOs and siRNA molecules making them safer and more stable *in vivo* with enhanced potency and efficacy. Delivery of ASOs and siRNAs to the brain is one of the biggest hurdles in development of RNA therapeutics for CNS disorders and requires bypassing of BBB by IT or ICV injections. Thus, further advances in drug delivery methods to the CNS are required to increase patient comfort and clinical ease of drug administration. On the other hand, increased stability of chemically modified RNA drugs with prolonged duration of action may create potential adverse effects, if the treatment is not tolerated by the patient. Reversal of siRNA and ASO activity has already been demonstrated experimentally[123,124], but further research is needed in discovery of RNA drugs for CNS disorders as unintentional side effects can have serious ramifications on e.g. cognition and general well-being of the patients. Nevertheless, the successful approval of seven RNA drugs by the FDA and EMA for treatment of SMA, DMD, FAP, underscores the high potential of RNA-based therapeutics as a modality for generating disease-modifying drugs for a wide variety of neurological diseases and neuromuscular disorders.
